# Atomically precise nanoclusters with reversible isomeric transformation for rotary nanomotors

**DOI:** 10.1038/s41467-020-19789-4

**Published:** 2020-11-26

**Authors:** Zhaoxian Qin, Jiangwei Zhang, Chongqing Wan, Shuang Liu, Hadi Abroshan, Rongchao Jin, Gao Li

**Affiliations:** 1 State Key Laboratory of Catalysis, Dalian Institute of Chemical Physics, Chinese Academy of Sciences, 116023 Dalian, China; 2 Beijing Key Laboratory for Optical Materials and Photonic Devices, Department of Chemistry, Capital Normal University, 100048 Beijing, China; 3 University of Chinese Academy of Sciences, 100049 Beijing, China; 4 Department of Chemistry, Carnegie Mellon University, Pittsburgh, PA 15213 USA

**Keywords:** Chemical synthesis, Nanoscale materials

## Abstract

Thermal-stimuli responsive nanomaterials hold great promise in designing multifunctional intelligent devices for a wide range of applications. In this work, a reversible isomeric transformation in an atomically precise nanocluster is reported. We show that biicosahedral [Au_13_Ag_12_(PPh_3_)_10_Cl_8_]SbF_6_ nanoclusters composed of two icosahedral Au_7_Ag_6_ units by sharing one common Au vertex can produce two temperature-responsive conformational isomers with complete reversibility, which forms the basis of a rotary nanomotor driven by temperature. Differential scanning calorimetry analysis on the reversible isomeric transformation demonstrates that the Gibbs free energy is the driving force for the transformation. This work offers a strategy for rational design and development of atomically precise nanomaterials via ligand tailoring and alloy engineering for a reversible stimuli-response behavior required for intelligent devices. The two temperature-driven, mutually convertible isomers of the nanoclusters open up an avenue to employ ultra-small nanoclusters (1 nm) for the design of thermal sensors and intelligent catalysts.

## Abstract (web summary)

Atomically precise metal nanoclusters are an emerging class of precision nanomaterials and hold potential in many applications. Here, the authors devise a [Au_13_Ag_12_(PPh_3_)_10_Cl_8_]^+^ nanocluster with two conformational isomers that can reversibly convert in response to temperature, and hence acts as a rotary nanomotor.

## Introduction

Stimuli-responsive materials are at the forefront of technological innovations to fabricate the next-generation devices with “intelligent” performance^1–3^. To meet the self-activation and -deactivation functionality requirements for smart materials, the stimuli-responsive behavior should be reversible. This can be satisfied by utilization of precise structural and phase transitions^4–8^. In particular, reversible conformational isomerism, a fundamental concept in molecular sciences that is commonly observed in organic molecules, offers a unique platform to prepare rotary motors at the nanoscale with atomistic precision.

Despite recent advances in controllable synthesis and structure determination of atomically precise nanoclusters^9–18^, the concept of reversible conformational isomerization for metal nanoclusters has not yet been explored. To date, irreversible structural isomerization of atomically precise nanoclusters has been reported in a few cases^19–24^. One case pertains to Au_42_(TBBT)_26_ (TBBT = 4-tert-butylbenzenethiolate) in the form of fcc and non-fcc frameworks that were synthesized via two different procedures^19^. However, the two isomeric structures of Au_42_ are “static” and cannot be reversibly transformed^19^. The same is true to the clusters prepared by Teo et al.^20–23^. One directional structural conversion from metastable Au_38_(SC_2_H_4_Ph)_24_ to thermodynamically stable biicosahedral Au_38_(SC_2_H_4_Ph)_24_ was reported to occur under thermal conditions, but unfortunately the reverse transformation could not occur^24^. Finally, while reversible transformation between Au_28_(SC_6_H_11_)_20_ and Au_28_(SPhC_4_H_9_)_20_ nanoclusters was realized by Chen et al., this case involves different surface-protecting ligands (-SC_6_H_11_ vs. -SC_4_H_9_), so it does not strictly meet the definition of isomeric transformation^25^ and cannot be utilized for construction of nanomotors. Therefore, breakthroughs are still needed to precisely modulate the bonding structure of ultra-small nanoparticles to make their frameworks flexible for a facile molecular rotary nanomotor.

**Fig. 1:**
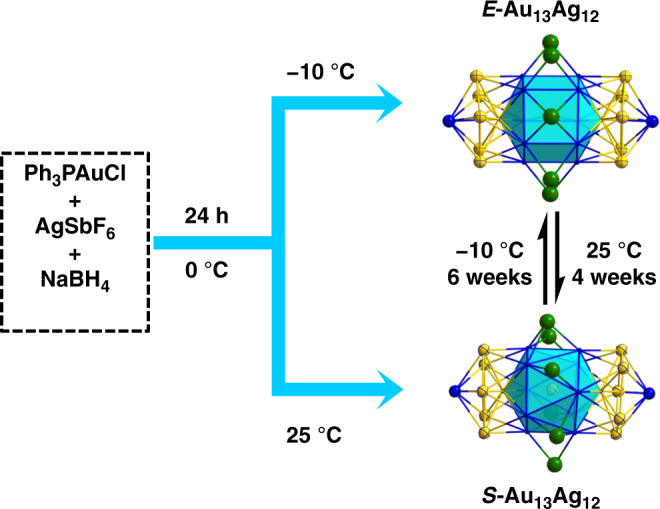
Two isomers of the [Au_13_Ag_12_(PPh_3_)_10_Cl_8_]^+^(SbF_6_)ˉ nanocluster with thermally responsive transformation (h = hour). *E-* eclipsed configuration, *S-* staggered configuration. Color code: Au: yellow; Ag: blue; Cl: green (C, H, P, and some Cl are omitted for clarity).

Herein, we report the discovery of a stimuli-responsive nanocluster that shows reversible conformational isomerism. The [Au_13_Ag_12_(PPh_3_)_10_Cl_8_]^+^(SbF_6_)ˉ nanocluster, hereafter abbreviated as Au_13_Ag_12_, presents two isomeric forms that can be selectively obtained by simply controlling the temperature (Fig. 1), hence forming the basis of a rotary nanomotor controlled by temperature. Through a combined approach of experiments, we have investigated the structure of the nanocluster and its stimuli-responsive isomerization. The isomers possess distinct optical properties, which can be utilized for advancing intelligent sensors, catalysts, and controlled drug release in biomedical applications.

## Results

### Synthesis and characterization of Au_13_Ag_12_ isomers

**Fig. 2:**
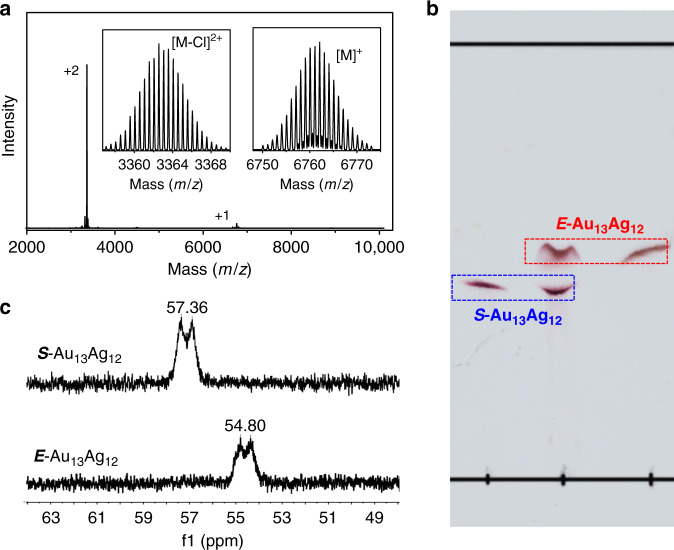
Characterization of the Au_13_Ag_12_ nanoclusters. **a** Positive-mode ESI mass spectrum of the as-synthesized Au_13_Ag_12_ nanocluster. (M = Au_13_Ag_12_(PPh_3_)_10_Cl_8_) **b** Thin-layer chromatography separation of the Au_13_Ag_12_ using CH_2_Cl_2_/CH_3_OH (2:1, v/v) as an eluent. **c** ^31^P NMR spectra of the *S*-Au_13_Ag_12_ and *E*-Au_13_Ag_12_ in CD_2_Cl_2_.

The Au_13_Ag_12_ nanocluster was synthesized by reduction of a mixed solution of Ph_3_PAuCl and AgSbF_6_ using NaBH_4_ in an ice bath (see Supplementary Methods). The nanoclusters were analyzed by electrospray ionization mass spectrometry (ESI-MS) in a positive mode. A weak mass peak at *m*/*z* = 6762.10 and an intense one at *m*/*z* = 3362.56 were observed in the mass spectrum (Fig. 2a), with the spacing of their isotope patterns being 1 and 0.5 (Fig. 2a, inset), hence, 1+ and 2+ charges, respectively. The two mass peaks correspond to [Au_13_Ag_12_(PPh_3_)_10_Cl_8_]^+^ (theoretical *m*/*z*: 6761.46 Da, deviation: 0.62 Da) and [Au_13_Ag_12_(PPh_3_)_10_Cl_7_]^2+^ (theoretical *m*/*z*: 3363.00 Da, deviation: −0.44 Da), respectively, with the latter (2+ ion) being formed via loss of a Cl^−^ from the intact 1+ cluster during the ESI-MS analysis. X-ray crystallographic analysis (vide infra) confirmed homogeneous [Au_13_Ag_12_(PPh_3_)_10_Cl_8_]^+^, with no observation of [Au_13_Ag_12_(PPh_3_)_10_Cl_7_]^2+^. It is worth noting that loss of a Cl^−^ ligand often occurs in ESI-MS analysis^26^. Therefore, the molecular formula of the synthesized nanoclusters is [Au_13_Ag_12_(PPh_3_)_10_Cl_8_]^+^, and the product is highly pure.

Surprisingly, two species were identified in the Au_13_Ag_12_ product by thin-layer chromatography (TLC) with CH_2_Cl_2_/CH_3_OH (2:1, v/v) as the eluent^27^. The two distinct bands (Fig. 2b) indicate two isomers of Au_13_Ag_12_ since they share the same composition, hereafter denoted as *E*- and *S*-Au_13_Ag_12_ (vide infra). The bands were cut out and extracted with CH_2_Cl_2_, and their optical spectra indeed show different profiles of the two isomers of Au_13_Ag_12_ (see Supplementary Fig. 1). The ultraviolet–visible (UV-vis) spectrum of the mixed isomers exhibit peaks at 324, 361, 420, 500, and 656 nm. After TLC separation, the *E*-Au_13_Ag_12_ nanocluster shows three peaks at 361, 418, and 497 nm, whereas the *S*-Au_13_Ag_12_ isomer shows four peaks at 330, 423, 510, and 657 nm. The energy gaps of the *S*- and *E*-Au_13_Ag_12_ isomers are 1.72 and 2.17 eV, respectively (see Supplementary Fig. 1, inset). Interestingly, the pure *S*- and *E*-Au_13_Ag_12_ isomers show different ^31^P nuclear magnetic resonance (NMR) signals: 54.80 ppm for *E*-Au_13_Ag_12_ and 57.36 ppm for *S*-Au_13_Ag_12_ using Au(PPh_3_)Cl (^31^P NMR: 33.13 ppm) as the internal reference (Fig. 2c and see Supplementary Fig. 2). It demonstrates that the *E*-Au_13_Ag_12_ and *S*-Au_13_Ag_12_ nanoclusters have different structures (vide infra).

### Crystal structures

**Fig. 3:**
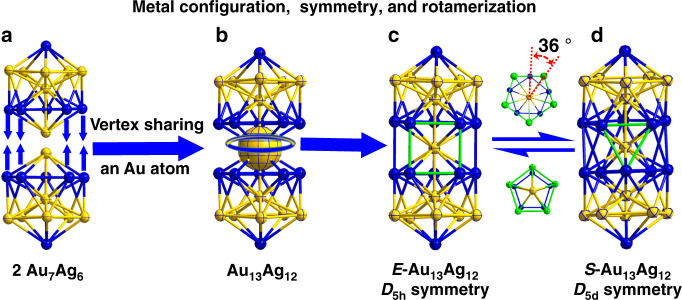
Configuration of the Au_13_Ag_12_ nanocluster. **a**, **b** Two icosahedral Au_7_Ag_6_ units share one common vertex of Au to form the Au_13_Ag_12_ nanocluster. **c** Metal configuration of *E*-isomer. **d** Metal configuration of *S*-isomer. Color code: Au: yellow; Ag: blue. Other atoms are omitted for clarity.

Furthermore, we found that keeping a fresh solution of the Au_13_Ag_12_ nanocluster containing both isomers at 25 °C for 4 weeks led to a solution containing only the *S*-Au_13_Ag_12_ isomer. In contrast, under −10 °C the same isomeric mixture completely transforms to the *E*-Au_13_Ag_12_ isomer after 6 weeks. Crystallizations of the *S*-Au_13_Ag_12_ and *E*-Au_13_Ag_12_ isomers were performed via a vapor diffusion method, followed by X-ray diffraction analysis. The total structures of *S*-Au_13_Ag_12_ and *E*-Au_13_Ag_12_ are displayed in Supplementary Figs. 3 and 4. For the core frameworks, each isomer is composed of two icosahedral Au_7_Ag_6_ units fused together by sharing a common vertex of Au (Fig. 3a). The two Au_5_ pentagons at the ends of the rod are ligated by ten phosphine ligands. Two Cl ligands bind with two apical Ag atoms. Six Cl ligands bridge the two icosahedra via bonding with 10 equatorial Ag atoms (see Supplementary Fig. 5).

**Fig. 4:**
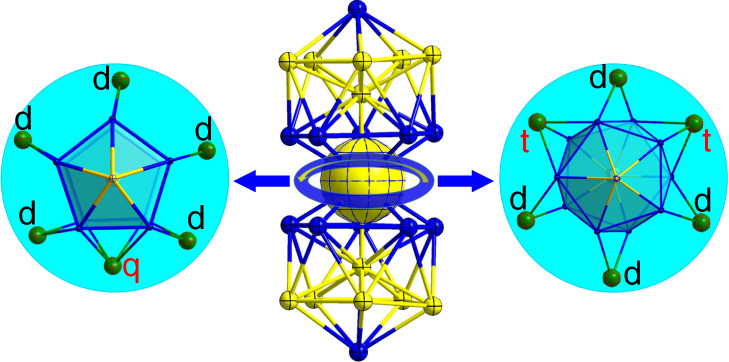
Structures of *E*- and *S*-Au_13_Ag_12_ isomers from the top view. Doubly (d), triply (t), and quadruply (q) binding patterns of six equatorial Cl ligands bridging the two icosahedra. Color codes: Au: yellow; Ag: blue; Cl: green.

The central Au atom (enlarged in Fig. 3b) connects the two Au_7_Ag_6_ icosahedra and serves as the pivot for the rotamerization of the metal configuration of the Au_13_Ag_12_. At −10 °C, the two Au_7_Ag_6_ units are in an eclipsed (*E*) configuration with *D*_5h_ symmetry (i.e., *E*-Au_13_Ag_12_, Fig. 3c), whereas at 25 °C the two Au_7_Ag_6_ units rotate by about 36° to form a staggered (*S*) configuration with *D*_5d_ symmetry (*S*-Au_13_Ag_12_, Fig. 3d). We note that, at the lower temperature (−10 °C), the Au_13_Ag_12_ nanocluster prefers to adapt the configuration with a higher symmetry. The six chloride ligands—which bridge the two Au_7_Ag_6_ units—serve as the “belt” to drive the rotamerization, Fig. 4. In the *E*-Au_13_Ag_12_ isomer, there are five Cl ligands with each being bonded with two silver atoms (d-mode). For the same isomer, there is also a Cl ligand that is bonded with four silver atoms (q-mode). In the *S*-Au_13_Ag_12_ isomer, four Cl ligands are in the d-mode bonding, and two Cl ligands are each bonded with three silver atoms (t-mode).

### Reversible transformation between the *E*- and *S*- isomer

**Fig. 5:**
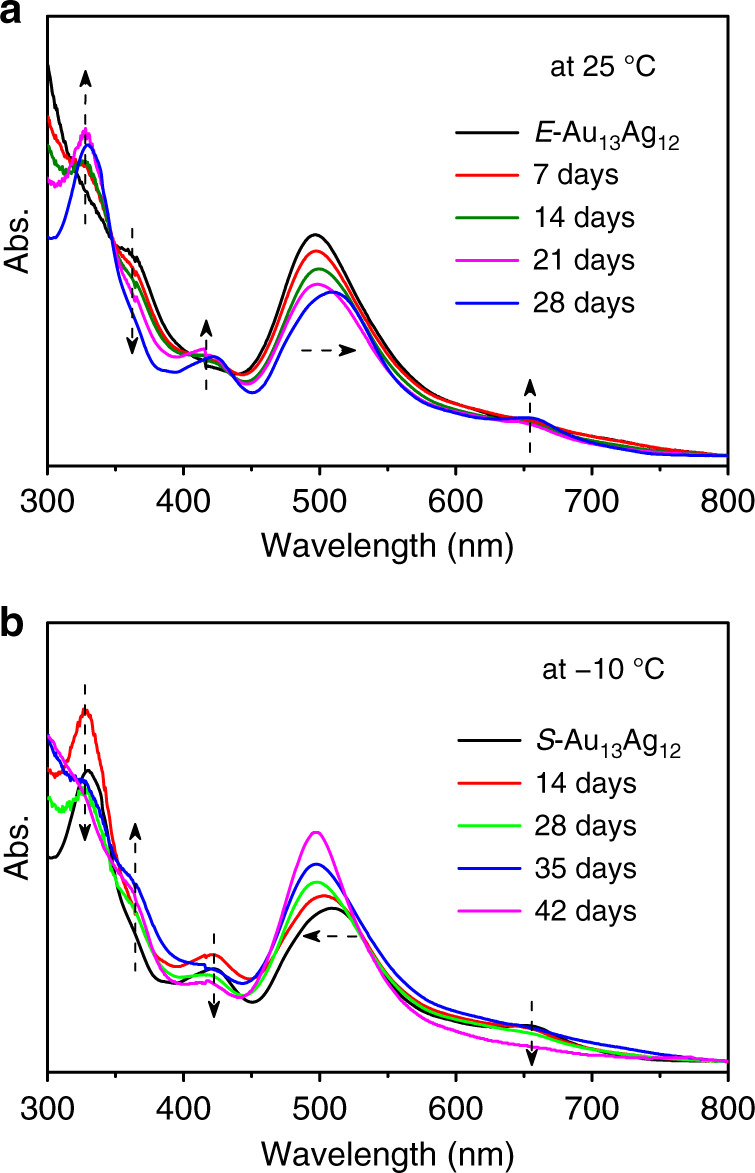
UV-vis spectral evolution for the isomeric transformation of the nanoclusters. **a** *E*-Au_13_Ag_12_ conversion to *S*-Au_13_Ag_12_ at 25 °C. **b** The as-obtained *S*-Au_13_Ag_12_ conversion to *E*-Au_13_Ag_12_ at −10 °C.

The *E*-Au_13_Ag_12_ and *S*-Au_13_Ag_12_ isomers can be 100% selectively obtained at −10 and 25 °C, respectively. To test the reversibility of the isomeric transformation, we monitored the process by UV-vis absorption spectroscopy using the crystal sample of *E*-Au_13_Ag_12_ nanoclusters as the starting material. We first studied the *E*-Au_13_Ag_12_ nanocluster in a dichloromethane solution at 25 °C. The characteristic peak at 361 nm gradually decreased over time and simultaneously peaks at 330, 423, and 657 nm gradually increased. The peak at 418 nm also red-shifted to 510 nm during a period of 28 days, Fig. 5a. The ^31^P NMR test of the sample gave a doublet-splitting peak centered at 57.36 ppm (see Supplementary Figs. 6 and 7). These results indicate that the *E*-Au_13_Ag_12_ isomer slowly converts to the *S*-Au_13_Ag_12_ isomer at 25 °C. We next studied the transformation of the as-obtained *S*-Au_13_Ag_12_ isomer at −10 °C. The characteristic peaks at 330 and 657 nm disappeared after 42 days. Meanwhile, the peak at 361 nm increased and the peak at 510 nm blue-shifted to 418 nm, demonstrating that the *S*-Au_13_Ag_12_ isomer transforms to the *E*-Au_13_Ag_12_ counterpart (Fig. 5b). This is further supported by ^31^P NMR analysis, as the NMR peak appears at 54.80 ppm (see Supplementary Figs. 8 and 9). Of note, no byproduct was formed during the reversible transformation, evidenced by the ESI-MS tests (see Supplementary Figs. 10 and 11). These results imply that both isomers are stable, and the isomeric transformation process is reminiscent of typical phase transitions.

**Fig. 6:**
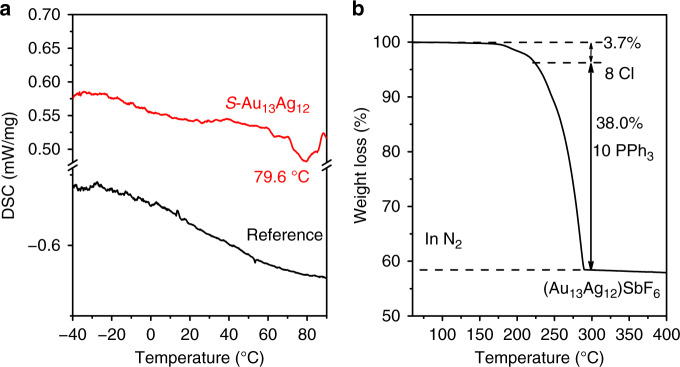
Thermal analysis of the Au_13_Ag_12_ nanoclusters. **a** DSC analysis for the conversion of the *S*-Au_13_Ag_12_ to *E*-Au_13_Ag_12_ under a N_2_ atmosphere. **b** TGA test of the Au_13_Ag_12_ nanoclusters under a N_2_ atmosphere.

Differential scanning calorimeter (DSC) method was used to investigate the isomeric transformation process and the corresponding enthalpy^28^. Starting from the *S*-Au_13_Ag_12_ isomer, a negative peak centered at 79.6 °C was observed during the DSC test, which points to endothermicity of the isomeric transformation of the *S*-Au_13_Ag_12_ to *E*-Au_13_Ag_12_ (Fig. 6a). Both UV-vis and ^31^P NMR analyses indicate that the *S*-Au_13_Ag_12_ isomer was partially converted to the *E*-Au_13_Ag_12_ isomer (see Supplementary Figs. 12 and 13). Furthermore, thermogravimetric analysis (TGA) revealed that the removal of Cl ligands from the nanoclusters occur at 175 °C (weight loss of ~3.7 wt%, consistent with the expected value of 4.0 wt%), Fig. 6b. Subsequently, the phosphine ligands began to desorb at ~210 °C, consistent with the desorption temperature reported for the phosphine-protected Au nanocluster^28,29^. The TGA results indicate that the Au_13_Ag_12_ nanoclusters are intact below 100 °C (see Supplementary Figs. 14 and 15); thus the observed endothermic process in the DSC analysis is solely associated with the isomeric transformation, rather than with any ligand loss.

Owning to the higher symmetry of the *E*-Au_13_Ag_12_ (*D*_5h_) than the *S*-Au_13_Ag_12_ (*D*_5d_), the nanocluster is a potential prototype of thermal molecular motor where Gibbs free energy serves as the driving force. In a previous work, Teo et al.^20–23^ synthesized two isomers of Au_13_Ag_12_(PPh_3_)_10_Br_8_ nanoclusters; however, no isomeric rotamerization was observed, which can be attributed to the stronger Ag–Br bonds than Ag–Cl. Our study shows that employing the –Cl ligand, instead of –Br, leads to higher flexibility on Ag–halide bonds, which is essential and critical to achieve a more flexible cluster framework for the thermally responsive, reversible transformation between the *E*-Au_13_Ag_12_ and *S*-Au_13_Ag_12_ isomers. Such a Cl-ligand-induced rotary nanomotor is simulated based on the experimental structures (see Supplementary Movie 1). For practical applications, the temperature will serve as the driving force.

In summary, two isomers of the biicosahedral [Au_13_Ag_12_(PPh_3_)_10_Cl_8_]^+^ (counterion: [SbF_6_]^−^) nanocluster (i.e., *S*-Au_13_Ag_12_ and *E*-Au_13_Ag_12_) are discovered, and these two isomers are reversibly transformable by controlling the temperature. The metal configuration of the *E*-Au_13_Ag_12_ isomer possesses a higher symmetry (*D*_5h_) than that of the *S*-Au_13_Ag_12_ isomer (*D*_5d_) and is preferably formed at low temperature (−10 °C). As the temperature increases to 25 °C, the *S*-Au_13_Ag_12_ isomer (lower symmetry) is exclusively formed. This study shows that the alloying and ligand engineering (i.e., Ag–halide bond) provide a rational strategy to make the framework of metal nanoclusters more flexible for achieving the conformational isomerism, which has the potential to be applied in designing intelligent molecular engines with Gibbs free energy as the driving force of the activity.

## Methods

### Synthesis of Au_13_Ag_12_ nanoclusters

Au(I)PPh_3_Cl (25 mg, dissolved in 2 mL chloromethane/methanol with *v*:*v* = 1:1) was mixed with AgSbF_6_ (17.2 mg, dissolved in 2 mL chloromethane/methanol with *v*:*v* = 1:1). The solution was stirred in dark and air atmosphere. Then the solution was cooled using an ice bath for 30 min, followed by dropwise addition of NaBH_4_ solution (2 mg, dissolved in 4 mL ice cold methanol). The mixture was kept in the dark and stirred for another 24 h. Next, the temperature was increased slowly to 25 °C. The mixture was then dried via vacuum evaporation and washed with hexane (2 × 1 mL), leaving a black solid. Finally, the black solid was dissolved in 2 mL dichloromethane/methanol (*v*:*v* = 1:1) and centrifuged at 10,000 rpm for 5 min. Red, plate-like crystals of *E*-Au_13_Ag_12_ and *S*-Au_13_Ag_12_ were obtained via slow diffusion of diethyl ether into the cluster solution over 6 weeks at −10 or 25 °C. The yields are 26.0% for *E*-Au_13_Ag_12_ and 34.9% for *S*-Au_13_Ag_12_ based on the consumption of Ph_3_PAuCl.

## Supplementary information

Supplementary Information

Description of Additional Supplementary Files

Supplementary Movie 1

## Data Availability

The X-ray crystallographic coordinates for structures reported in this study have been deposited at the Cambridge Crystallographic Data Centre (CCDC), under deposition numbers 1888164–1888165. These data can be obtained free of charge from The Cambridge Crystallographic Data Centre via www.ccdc.cam.ac.uk/data_request/cif. The datasets generated and/or analyzed during this study are available from the corresponding author upon reasonable request.

## References

[1] Petermayer C, Dube H (2018). Indigoid photoswitches: visible light responsive molecular tools. Acc. Chem. Res..

[2] Wei P (2018). Multiple yet controllable photoswitching in a single AIEgen system. J. Am. Chem. Soc..

[3] Zhang B (2018). Thermally-induced reversible structural isomerization in colloidal semiconductor CdS magic-size clusters. Nat. Commun..

[4] Fan X, Wang J, Wu K, Zhang L, Zhang J (2019). Isomerism in titanium-oxo clusters: molecular anatase model with atomic structure and improved photocatalytic activity. Angew. Chem. Int. Ed..

[5] Wang H, Zhang J, Xie Z (2017). Reversible photothermal isomerization of carborane-fused azaborole to borirane: synthesis and reactivity of carbene-stabilized carborane-fused borirane. Angew. Chem. Int. Ed..

[6] Sun J (2018). Stimuli-directed dynamic reconfiguration in self-organized helical superstructures enabled by chemical kinetics of chiral molecular motors. Adv. Sci..

[7] Zhang J (2016). The proton-controlled synthesis of unprecedented diol functionalized Anderson-type POMs. Chem. Commun..

[8] Zhang J (2015). Unprecedented χ isomers of single-side triolfunctionalized Anderson polyoxometalates and their proton-controlled isomer transformation. Chem. Commun..

[9] Konishi K, Iwasaki M, Shichibu Y (2018). Phosphine-ligated gold clusters with core+exo geometries: unique properties and interactions at the ligand−cluster interface. Acc. Chem. Res..

[10] Cook AW, Hayton TW (2018). Case studies in nanocluster synthesis and characterization: challenges and opportunities. Acc. Chem. Res..

[11] Lei Z, Wan X-K, Yuan S-F, Guan Z-J, Wang Q-M (2018). Alkynyl approach toward the protection of metal nanoclusters. Acc. Chem. Res..

[12] Yao Q, Chen T, Yuan X, Xie J (2018). Toward total synthesis of thiolate-protected metal nanoclusters. Acc. Chem. Res..

[13] Zheng K (2019). Motif-mediated Au_25_(SPh)_5_(PPh_3_)_10_X_2_ nanorods with conjugated electron delocalization. Nano Res..

[14] Zhang J (2018). Diphosphine-induced chiral propeller arrangement of gold nanoclusters for singlet oxygen photogeneration. Nano Res..

[15] Nieto-Ortega B, Bürgi T (2018). Vibrational properties of thiolate-protected gold nanoclusters. Acc. Chem. Res..

[16] Chakraborty P, Nag A, Chakraborty A, Pradeep T (2019). Approaching materials with atomic precision using supramolecular cluster assemblies. Acc. Chem. Res..

[17] Zhou M (2019). Three-orders-of-magnitude variation of carrier lifetimes with crystal phase of gold nanoclusters. Science.

[18] Jin R, Zeng C, Zhou M, Chen Y (2016). Atomically precise colloidal metal nanoclusters and nanoparticles: fundamentals and opportunities. Chem. Rev..

[19] Zhuang S (2019). Fcc versus non-fcc structural isomerism of gold nanoparticles with kernel atom packing dependent photoluminescence. Angew. Chem. Int. Ed..

[20] Teo BK, Zhang H (1992). Molecular machines: molecular structure of [(p-Tol_3_P)_10_Au_13_Ag_12_Cl_8_](PF_6_)-a cluster with a biicosahedral rotorlike metal core and an unusual arrangement of bridging ligands.. Angew. Chem. Int. Ed..

[21] Teo BK, Shi XB, Zhang H (1991). Cluster of clusters. Structure of a novel cluster [(Ph_3_P)_10_Au_13_Ag_12_Br_8_](SbF_6_) containing an exact staggered-eclipsed-staggered metal configuration: evidence of icosahedral units as building blocks. J. Am. Chem. Soc..

[22] Teo, B. K., Shi, X. B. & Zhang, H. Cluster rotamerism of a 25-metal-atom cluster [(Ph_3_P)_10_Au_13_Ag_12_Br_8_]^+^ monocation: a molecular rotary unit. *J. Chem. Soc. Chem. Commun*., 1195–1196 (1992).

[23] Teo BK, Zhang H (1991). Cluster of clusters. Structure of a new 25-metal-atom cluster [(p-Tol_3_P)_10_Au_13_Ag_12_Cl_7_](SbF_6_)_2_ containing a nearly staggered-eclipsed-staggered metal configuration and five doubly bridging ligands. Inorg. Chem..

[24] Tian S (2015). Structural isomerism in gold nanoparticles revealed by X-ray crystallography. Nat. Commun..

[25] Chen Y (2016). Isomerism in Au_28_(SR)_20_ nanocluster and stable structures. J. Am. Chem. Soc..

[26] Song Y (2016). How a single electron affects the properties of the “non-superatom” Au_25_ nanoclusters. Chem. Mater..

[27] Liao L (2016). Quantitatively monitoring the size-focusing of Au nanoclusters and revealing what promotes the size transformation from Au_44_(TBBT)_28_ to Au_36_(TBBT)_24_. Anal. Chem..

[28] Molard Y (2016). Clustomesogens: liquid crystalline hybrid nanomaterials containing functional metal nanoclusters. Acc. Chem. Res..

[29] Liu C, Abroshan H, Yan C, Li G, Haruta M (2016). One-Pot synthesis of Au_11_(PPh_2_Py)_7_Br_3_ for the highly chemoselective hydrogenation of nitrobenzaldehyde. ACS Catal..

