# Supplementary material for: Atomically precise nanoclusters with reversible isomeric transformation for rotary nanomotors
Source: Nat Commun. 2020 Nov 26;11:6019. doi: 10.1038/s41467-020-19789-4 (PMC7693277; doi:10.1038/s41467-020-19789-4)
Supplement: Supplementary file 1 — Supplementary Information [file 41467_2020_19789_MOESM1_ESM.pdf]

Supplementary Information

Atomically Precise Nanoclusters with Reversible Isomeric Transformation for Rotary Nanomotors

Zhaoxian Qin,[†,‡,$,&] Jiangwei Zhang,[†,&] Chongqing Wan,[‡,*] Shuang Liu,[†] Hadi Abroshan,[#,*] Rongchao Jin,[#,*] and Gao Li[†,$,*]

[†]*State Key Laboratory of Catalysis, Dalian Institute of Chemical Physics, Chinese Academy of Sciences, Dalian 116023, China*

[‡]*Beijing Key Laboratory for Optical Materials and Photonic Devices, Department of Chemistry, Capital Normal University, Beijing 100048, China*

[$]*University of Chinese Academy of Sciences, Beijing 100049, China.*

[#]*Department of Chemistry, Carnegie Mellon University, Pittsburgh, PA 15213 USA*

[&] J.Z and Z.Q contributed equally.

* Correspondence: wancq@cnu.edu.cn (C.W.); hadi.abroshan@gmail.com (H.A.); gaoli@dicp.ac.cn (G.L.); rongchao@andrew.cmu.edu (R.J.)

EXPERIMENTAL METHODS

General materials

All chemicals, including the solvents, were commercially available as reagent grade and used as received without further purification. $AuPh_3PCl$ (98%), $AgSbF_6$ (99%), and $NaBH_4$ (99%) were purchased from Adamas-beta®. Ultrapure water was purified with a Barnstead Nanopure Di-water TM system. All glassware was thoroughly cleaned with aqua regia (37 wt% $HCl:HNO_3$ = 3:1 by volume), rinsed with copious Nano-pure water, and then dried in an oven prior to use.

Synthesis of $Au_{13}Ag_{12}$ nanoclusters

Au(I)PPh_3Cl (25 mg, dissolved in 2 mL chloromethane/methanol with v:v=1:1) was mixed with $AgSbF_6$ (17.2 mg, dissolved in 2 mL chloromethane/methanol with v:v=1:1). The solution was stirred in the dark and air atmosphere. Then, the solution was cooled using an ice bath for 30 min, followed by dropwise addition of $NaBH_4$ solution (2 mg, dissolved in 4 mL ice cold methanol). The mixture was kept in the dark and stirred for another 24 hrs. After that, the temperature was increased slowly to 25 ^{o}C. The mixture was then dried via vacuum evaporation, and washed with hexane (2×1 mL), leaving a black solid. Finally, the black solid was dissolved in 2 mL dichloromethane/methanol (v:v=1:1), and centrifuged at 10000 rpm for 5 min.

Red, plate-like crystals were obtained via slow vapor diffusion of ethyl ether into the cluster solution over weeks at -10 or 25 ^{o}C. At different temperatures, two different isomers were obtained. Yield: 6.7 mg, 26.0% for E-isomer, and 9.0 mg, 34.9% for S-isomer, based on Ph_3PAuCl.

General methods

UV-Vis spectra were measured in (dissolved in CH_2Cl_2/methanol (v:v=1:1)) by Shimadzu UV-2100S spectrophotometer. The mass spectra were obtained using an ion trap mass spectrometer (Thermofisher LTQ). Positive mode was chosen for the experiments (capillary voltage 33 V). The sample solution was infused into the ESI source at a flow rate of 300 μL min^{-1}. The sample was dissolved in dichloromethane (1 mg/mL) and then mixed 1:1 (v/v) with dry methanol. Thermal gravimetric analysis (TGA) was conducted with ~5 mg samples under a N_2 atmosphere (flow rate ~50 mL/min) on a STARe System (METTLER TOLEDO) at a heating rate of 10 °C/min. Differential scanning calorimeter (DSC) analysis was performed with ~8 mg samples on a NETZSCH DSC 204 with a N_2 flow (20 mL/min) in the range of -40 to 100 °C. ^{31}P NMR was performed on an AVANCE III NMR spectrometer (Bruker, 400 MHz). A sample of ~10 mg was dissolved in CD_2Cl_2 forming a saturated solution.

X-ray crystallographic analysis

Data reduction, cell refinement and experimental absorption correction were performed with the software package of Bruker APEX3. The structures were solved by intrinsic phasing methods by ShelXT[1] 2018 and refined against F^2 by full-matrix least-squares by ShelXL[2] 2018. All non-hydrogen atoms were refined anisotropically. Hydrogen atoms were

generated geometrically. All the calculations were carried out by the program package of program package ver 1.2.10[3].

Crystal data and structure refinements

Crystal data for [$Au_{13}Ag_{12}(PC_{18}H_{15})_{10}Cl_8$]($SbF_6$) (*S*-$Au_{13}Ag_{12}$), orthorhombic, P*bca*, a=27.8922 Å, b=26.3961 Å, c=52.2104 Å, V=38440 $Å^3$, Z=8, T=127 K, 32346 reflections measured, R_1=0.0844, wR_2=0.2014. Crystal data for [$Au_{13}Ag_{12}(PC_{18}H_{15})_{10}Cl_8$]($SbF_6$) (*E*-$Au_{13}Ag_{12}$), monoclinic, $P2_1/m$, a=16.632 Å, b=24.033 Å, c=29.956 Å, β=102.90°, V=11671 $Å^3$, Z=2, T=100 K, 23361 reflections measured, R_1= 0.0974, wR_2=0.2114.

CCDC-1888164-1888165 contain the supplementary crystallographic data for *S*-$Au_{13}Ag_{12}$ and *E*-$Au_{13}Ag_{12}$ in this paper. These data can be obtained free of charge from The Cambridge Crystallographic Data Centre (CCDC) via www.ccdc.cam.ac.uk/data_request/cif.

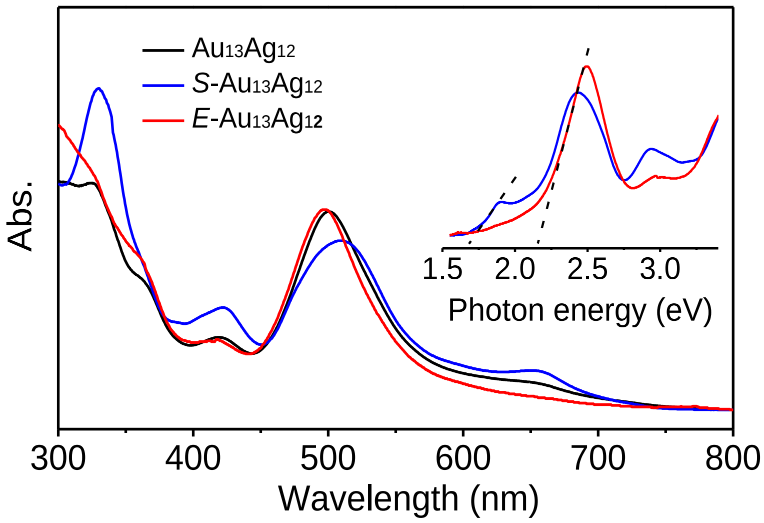

Supplementary Figure 1 UV-vis spectra of the *S*-$Au_{13}Ag_{12}$ (blue) and *E*-$Au_{13}Ag_{12}$ (red) in dichloromethane. Inset: spectra on the photon energy scale.

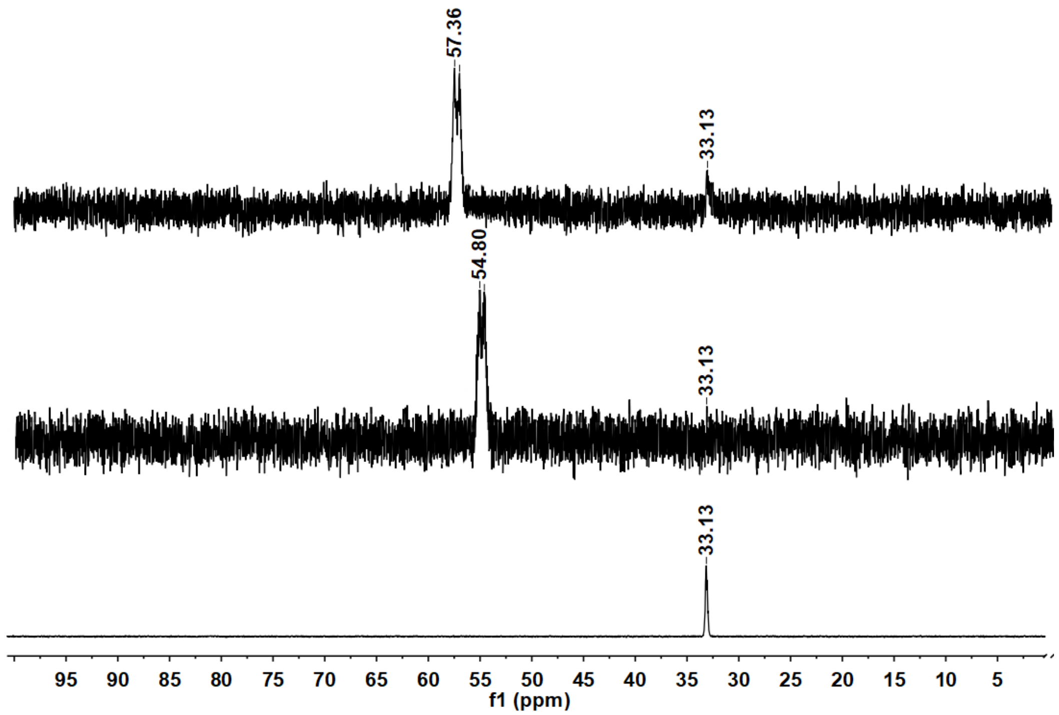

Supplementary Figure 2 ^{31}P NMR of the pure *S*-$Au_{13}Ag_{12}$ (upper line) and *E*-$Au_{13}Ag_{12}$ (middle line) crystal samples in CD_2Cl_2 solution using $Au(PPh_3)Cl$ (at 33.13 ppm, bottom line) as the internal reference.

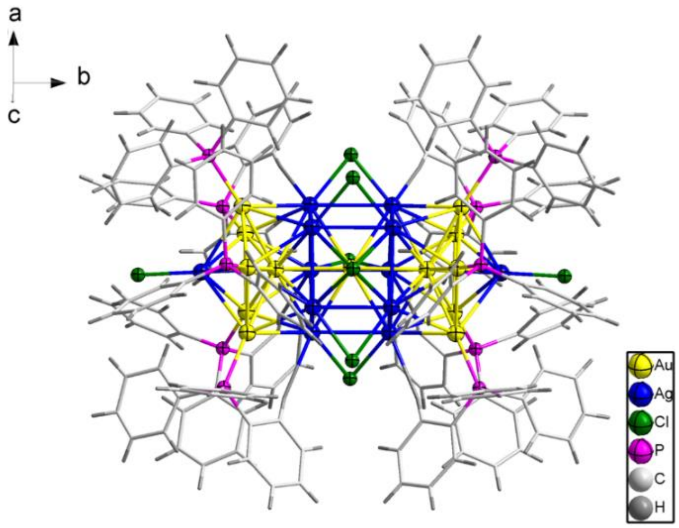

Supplementary Figure 3 Total structure of *E*-$Au_{13}Ag_{12}(PPh_3)_{10}Cl_8$ in ORTEP drawing.

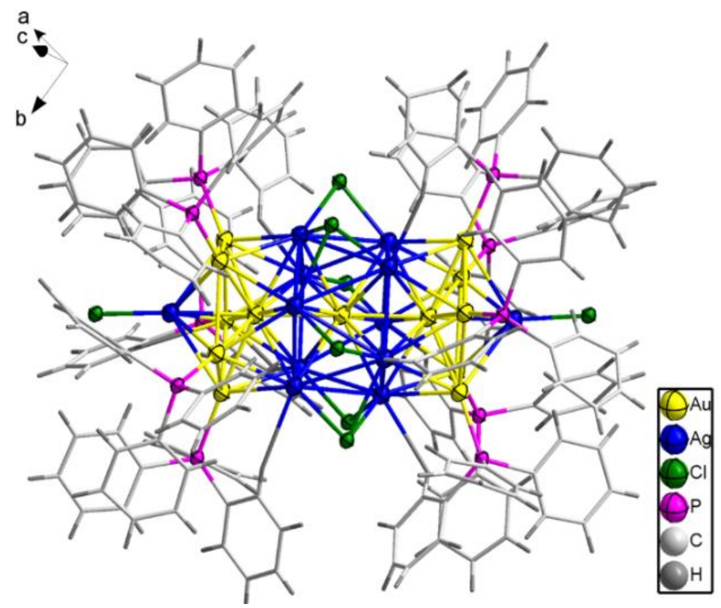

Supplementary Figure 4 Total structure of *S*-$Au_{13}Ag_{12}(PPh_3)_{10}Cl_8$ in ORTEP drawing.

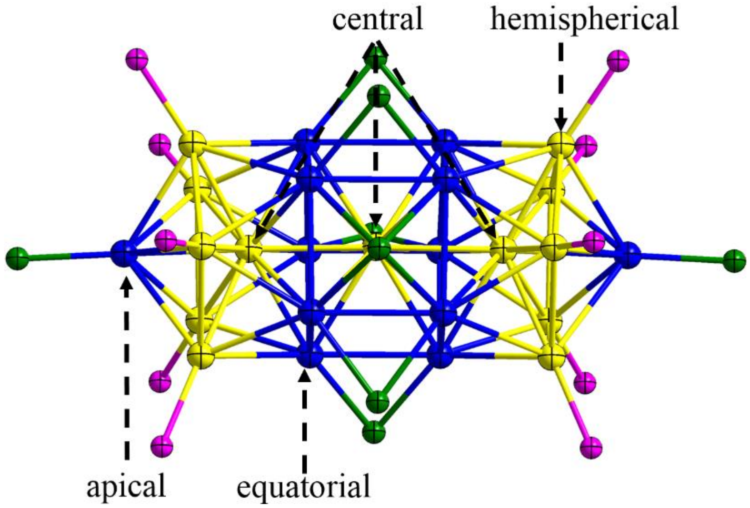

Supplementary Figure 5 Framework of the $Au_{13}Ag_{12}(PPh_3)_{10}Cl_8$ nanocluster.[4]

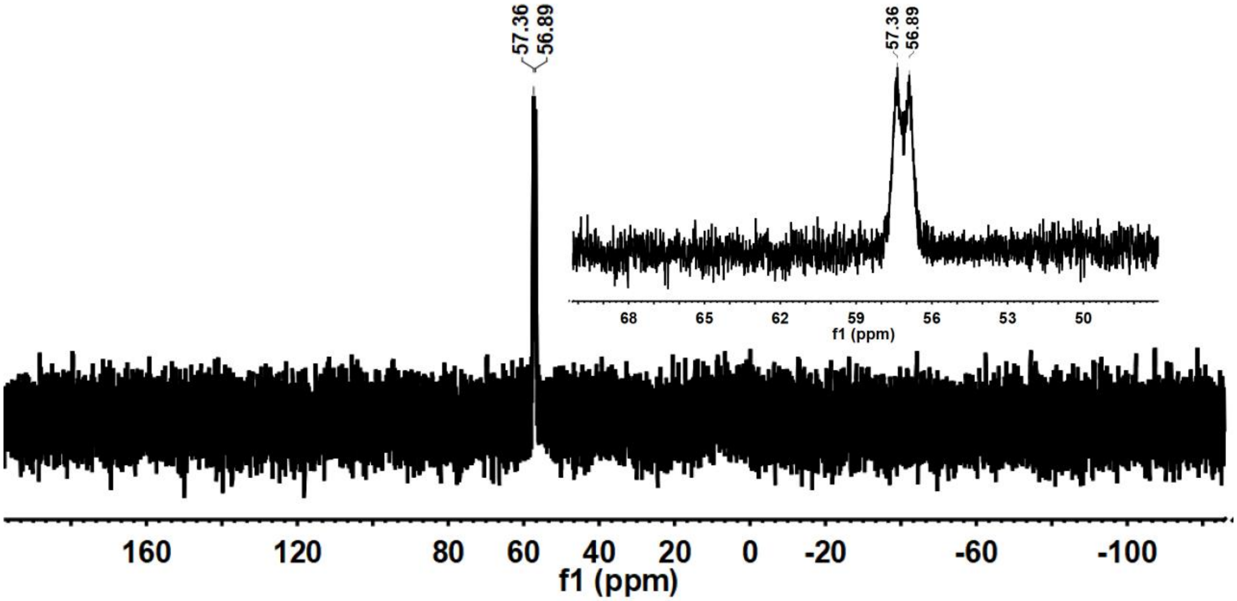

Supplementary Figure 6 ^{31}P NMR spectrum of the as-obtained *S*-$Au_{13}Ag_{12}$ after 28-day isomerization from *E*-$Au_{13}Ag_{12}$ to *S*-$Au_{13}Ag_{12}$. The single doublet peak found at 57.36 ppm implies the formation of pure *S*-$Au_{13}Ag_{12}$ clusters.

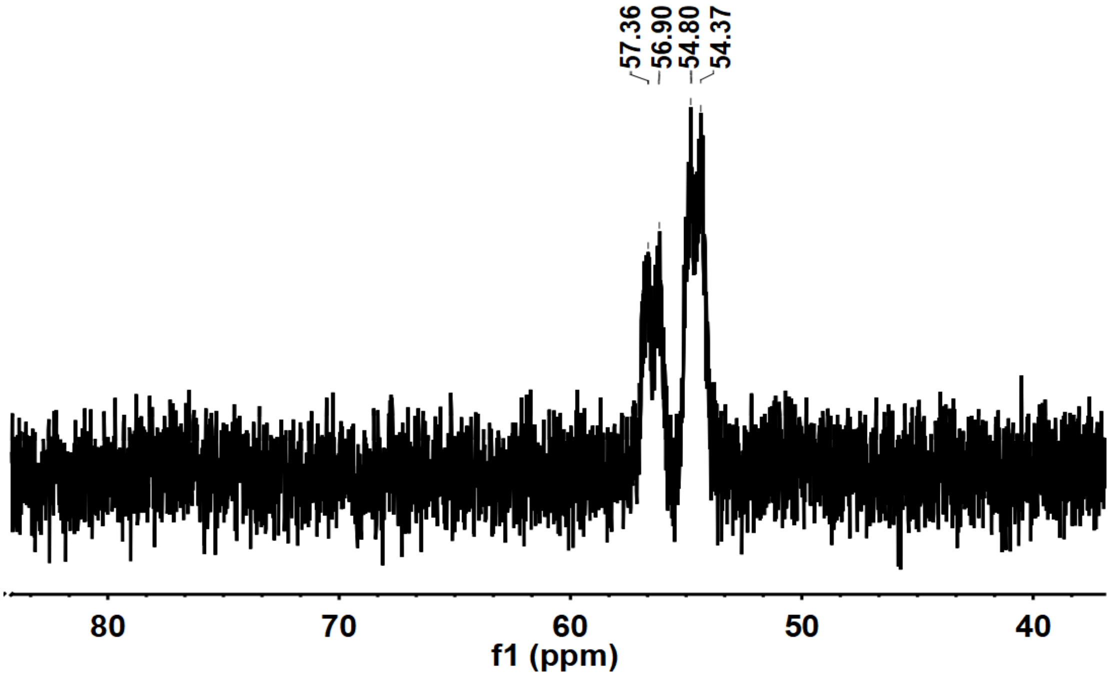

Supplementary Figure 7 ^{31}P NMR spectrum of the system after ~2 weeks for the conversion process from the *E*-$Au_{13}Ag_{12}$ isomer to the *S*-$Au_{12}Ag_{13}$ isomer. Two doublet peaks were found at 57.36 ppm and 54.80 ppm, suggesting the occurrence of conversion from the pure *E*-$Au_{13}Ag_{12}$ isomer to the *S*-$Au_{13}Ag_{12}$ isomer.

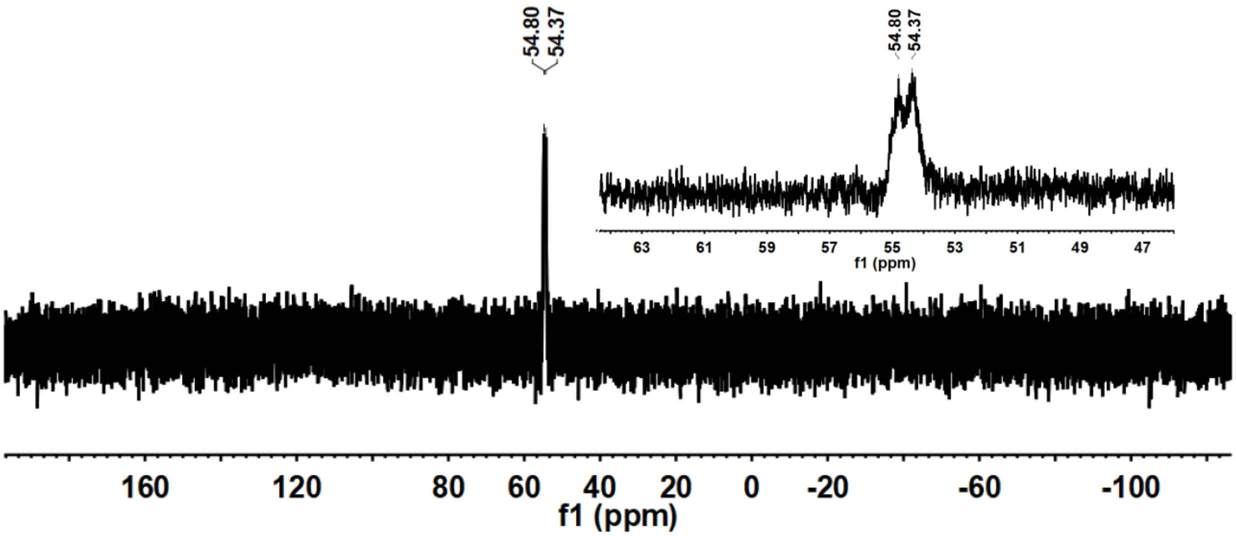

Supplementary Figure 8 ^{31}P NMR spectrum of the as-obtained *E*-$Au_{13}Ag_{12}$ clusters after 42-day isomerization from *S*-$Au_{13}Ag_{12}$ to *E*-$Au_{13}Ag_{12}$. The single doublet peak found at 54.80 ppm implies the formation of pure *E*-$Au_{13}Ag_{12}$ clusters.

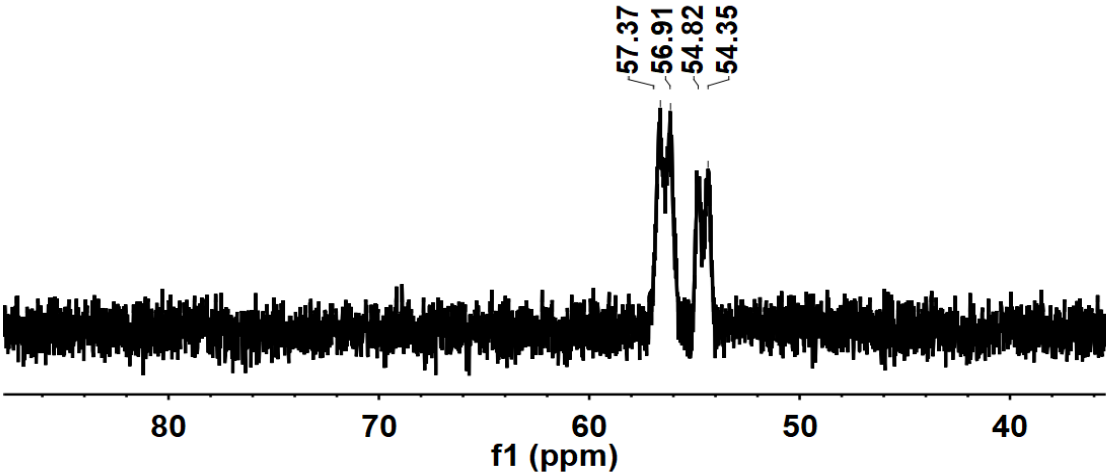

Supplementary Figure 9 ^{31}P NMR spectrum of the system after ~3 weeks for the conversion process from the *S*-$Au_{13}Ag_{12}$ isomer to the *E*-$Au_{12}Ag_{13}$ isomer. Two doublet peaks were found at 57.37 ppm and 54.82 ppm, illustrating the occurrence of conversion from the obtained pure *S*-$Au_{13}Ag_{12}$ isomer to the *E*-$Au_{13}Ag_{12}$ isomer.

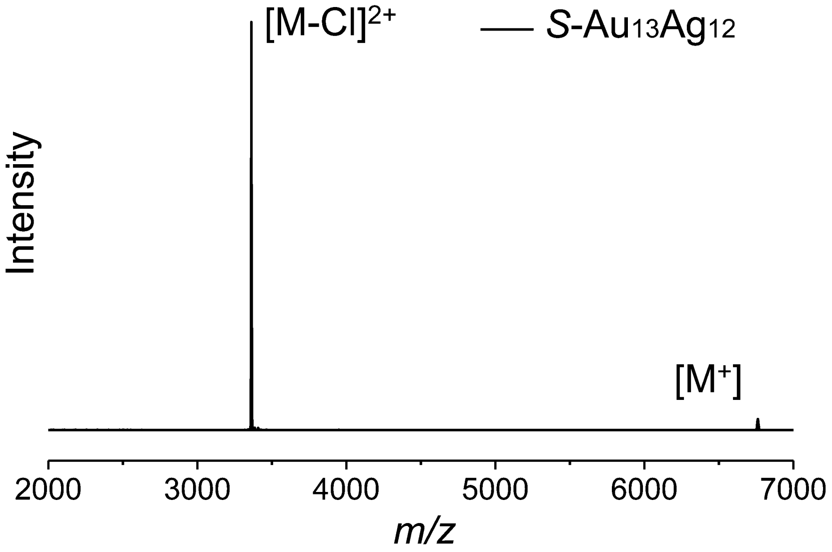

Supplementary Figure 10 ESI-MS of the as-obtained *S*-$Au_{13}Ag_{12}$ clusters which were formed after 28-day isomerization from *E*-$Au_{13}Ag_{12}$ to *S*-$Au_{13}Ag_{12}$.

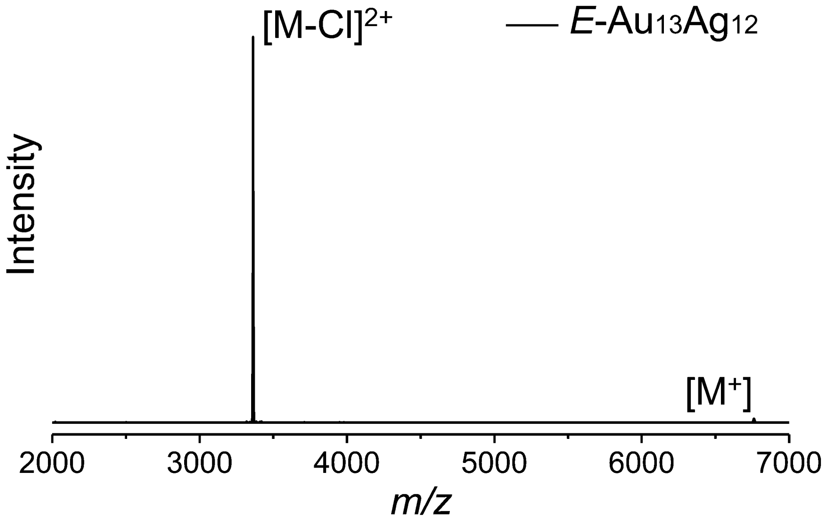

Supplementary Figure 11 ESI-MS of the as-obtained *E*-$Au_{13}Ag_{12}$ clusters after 42-day isomerization from *S*-$Au_{13}Ag_{12}$ to *E*-$Au_{13}Ag_{12}$.

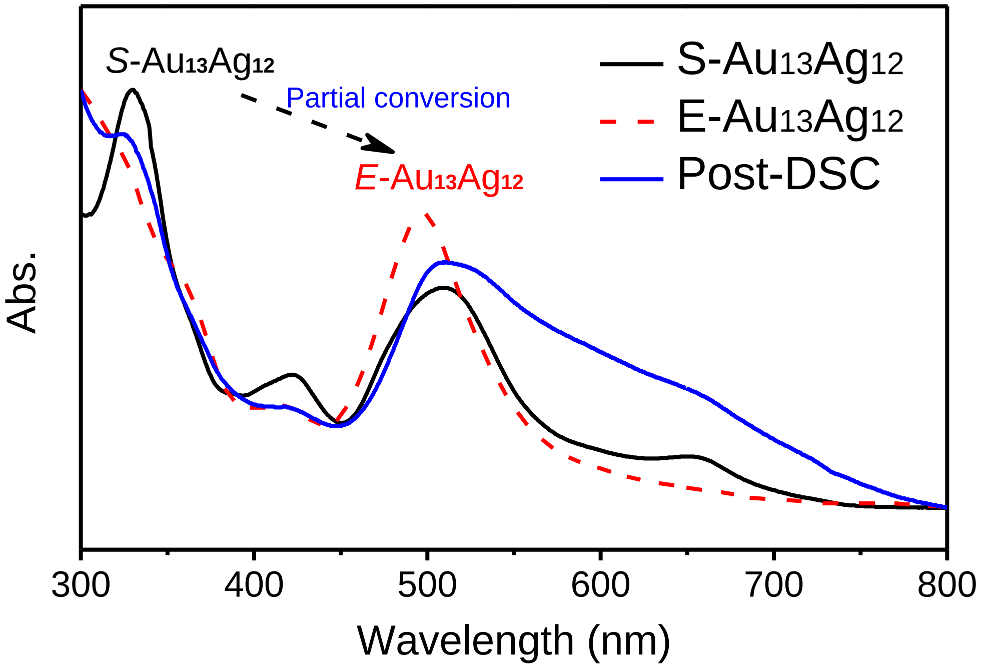

Supplementary Figure 12 UV-vis spectra of samples after the DSC experiment using *S*-$Au_{13}Ag_{12}$ isomer as the starting material.

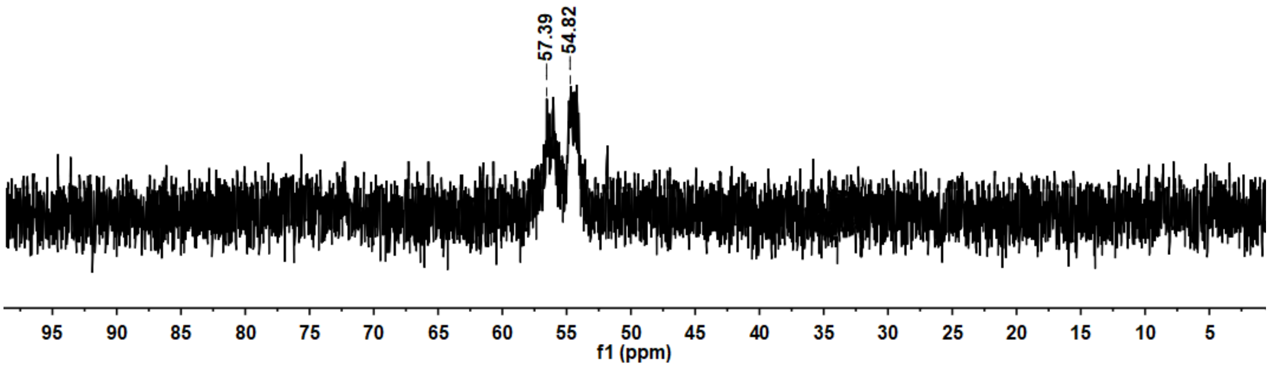

Supplementary Figure 13 ^{31}P NMR spectrum of the sample after the DSC experiment. Two peaks at ~57.39 and 54.82 ppm are found, indicating the presence of both *S*- and *E*-$Au_{13}Ag_{12}$ isomers.

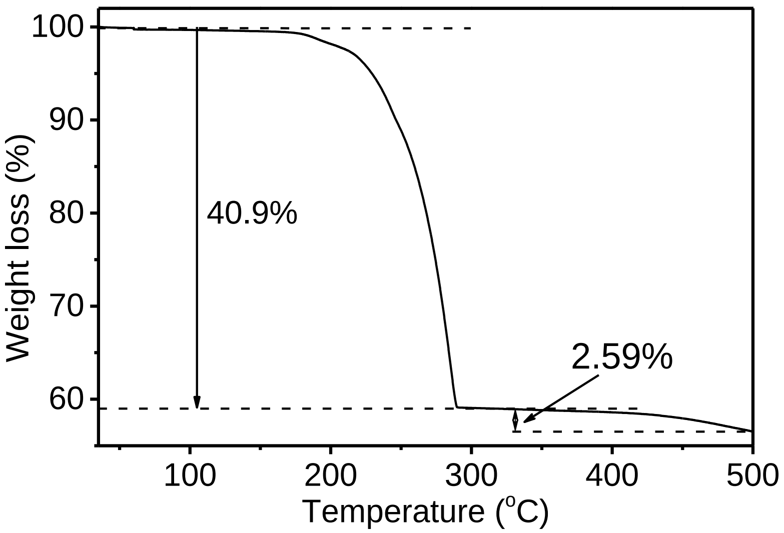

Supplementary Figure 14 TGA of $Au_{13}Ag_{12}$ sample under a N_2 atmosphere. The weight loss at ~250 °C is due to organic ligand loss. A second weight loss occurred at ~436 °C, which is assigned to be the removal of SbF_6 species.

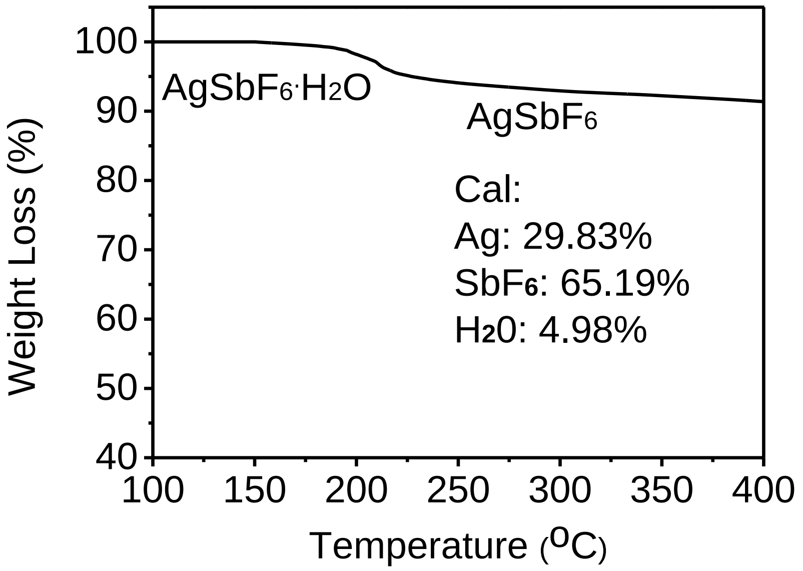

Supplementary Figure 15 TGA of $AgSbF_6{\cdot}H_2O$ under a N_2 atmosphere. The SbF_6 species was not lost below 400 °C, except the H_2O loss at ~200 °C.

References

(1) G. M. Sheldrick, *Acta Crystallogr. A* **71**, 3 (2015).
(2) G. M. Sheldrick, *Acta Crystallographica Section C* **71**, 3 (2015).
(3) O. V. Dolomanov, L. J. Bourhis, R. J. Gildea, J. A. K. Howard, H. Puschmann, *J. Appl. Crystallogr.* **42**, 339 (2009).
(4) K. Zheng, J. Zhang, D. Zhao, Y. Yang, Z. Li, G. Li, *Nano Res*. **12**, 501 (2019).
