# Supplementary material for: Atomically precise nanoclusters with reversible isomeric transformation for rotary nanomotors
Source: Nat Commun. 2020 Nov 26;11:6019. doi: 10.1038/s41467-020-19789-4 (PMC7693277; doi:10.1038/s41467-020-19789-4)
Supplement: Supplementary file 2 — Description of Additional Supplementary Files [file 41467_2020_19789_MOESM2_ESM.pdf]

Description of Additional Supplementary Files

File Name: Supplementary Movie 1
Description: Nanocluster-based rotary nanomotor
